# “That kind of changes things”: a meta-synthesis of the lived experiences of people with chronic heart disease

**DOI:** 10.1186/s12955-025-02423-6

**Published:** 2025-09-18

**Authors:** Lisa Nebel, Timothy Le Butt, Christian Sell, Christoph Herrmann-Lingen, Daniel Broschmann

**Affiliations:** 1 Department of Psychosomatic Medicine and Psychotherapy, University Medical Center Göttingen, Göttingen, Germany; 2 International Psychoanalytic University Berlin, Berlin, Germany; 3 German Center for Cardiovascular Research (DZHK), Partner site Göttingen, Göttingen, Germany

**Keywords:** Chronic heart disease, Quality of life, Lived experiences, Meta-Synthesis, IPA

## Abstract

**Aims:**

To explore the lived experiences of people with chronic heart disease (PCHD).

**Design:**

Systematic meta-synthesis.

**Methods:**

Following preregistration on PROSPERO, a systematic literature search was conducted in PubMed, PsycInfo, PsycArticles, and PSYNDEX between February 2023 and March 2024. Articles were assessed for eligibility based on predefined criteria and evaluated for methodological quality using a modified CASP tool. The results of the included primary studies were weighted according to their methodological quality and synthesized using Interpretative Phenomenological Analysis. The reporting adheres to PRISMA and ENTREQ guidelines.

**Results:**

The analysis of 43 articles revealed an overarching theme: “The broken flow of life.” This theme illustrates the disruption of normalcy, as perceived by patients through distinct dimensions. These dimensions are represented by the four subthemes: (1) I no longer feel safe in my body, (2) Suddenly, I have less of a future, (3) My identity feels shattered, and (4) My disease strains my relationships.

**Conclusions:**

The findings suggest that PCHD perceive their condition as a profound disruption of normalcy, affecting bodily, relational, and psychological dimensions that extend beyond established HRQOL measures.

**Implications:**

The findings have direct implications for the assessment of HRQOL in medicine. To adequately evaluate holistic treatments, a deeper understanding of how the disease affects life planning and future perspectives is essential. Consequently, established HRQOL measures may need to be extended both in content and temporal scope to capture these broader impacts.

**Supplementary Information:**

The online version contains supplementary material available at 10.1186/s12955-025-02423-6.

## Introduction

Heart disease is one of the most prevalent chronic conditions in Western societies, significantly impacting public health [1, 2]. Its prevalence is closely linked to unhealthy lifestyle habits, including diets high in fat and sodium, stress, or low levels of physical activity. As a result, the diagnosis of heart disease often necessitates profound lifestyle changes aimed at reducing mortality and morbidity. Such changes, along with the burden of the disease itself, can have a substantial impact on the lives of people with heart disease (PCHD). In clinical practice and research, the perspective of PCHD on their disease and treatment is, among other constructs, commonly assessed through health-related quality of life (HRQOL), a multidimensional construct that evaluates subjective experiences in terms of symptom clusters, capacities, and well-being across physical, social, and psychological dimensions [3].

While HRQOL provides valuable insights into the immediate impact of chronic heart disease on the subjective functioning and well-being, and has helped to shift the traditionally paternalistic medical dialogue towards a more patient-centered approach, it remains inherently limited to capturing the current state of health and well-being and does not fully reflect the longitudinal experience of living with a lifelong condition. Highlighting this limitation is particularly relevant for psychosomatic care, where the complex interplay between physical and mental health plays a crucial role, raising questions about how people can lead fulfilling lives under conditions of lifelong disease.

Established psychological and medical concepts that, alongside HRQOL, partially address the question of how to live well with disease include salutogenesis, which examines factors essential for maintaining health; well-being, which is often used interchangeably with quality of life (QOL); coping, which includes strategies that are intentionally (e.g., information-seeking) or unconsciously (e.g., denial) employed by peoples to manage their disease [4]; and recovery, which is traditionally conceptualized in medicine as the reduction of symptoms to pre-disease levels. However, in psychological discourse, particularly in the context of chronic mental illness where clinical recovery is not always attainable, the concept of personal recovery emphasizes the ability to live well with the disease, affirming life despite its challenges [5–7]. Similarly, posttraumatic growth (PTG) refers to the perceived personal growth following a traumatic event [8] such as receiving a lifelong diagnosis or experiencing an acute cardiac event. In medical research on acute cardiac events, perceived life changes are usually examined under the term of survivorship. Originating in cancer research, this term now broadly refers to the phase of a person’s life after the acute treatment of a serious illness, encompassing ongoing physical, psychological, and social issues related to having survived a life-threatening condition. These existential situations, which Jaspers termed ‘limit situations’, confront individuals with the limits of their existence, compelling them to reflect on the meaning of life [9].

These concepts, while valuable in highlighting various psychological and behavioral responses to illness, tend to emphasize specific aspects of the illness experience – coping focuses on strategies for managing the disease, salutogenesis emphasizes the promotion and maintenance of health, and PTG centers on the potential for personal development. While these perspectives contribute important insights, they only partially address the broader question raised earlier: what defines a good life for individuals living with a chronic condition such as heart disease? To complement and expand this perspective it is worthwhile to consider the philosophical discourse on the Good Life, which systematically analyzes what it means to live well. This discourse encompasses a range of theoretical approaches, including hedonism, which defines a good life by the quantity of positive experiences [10]; eudaimonia, which views the good life as the fulfillment of human capacities [11]; subjective preference theory, which posits that a good life is one where desires are satisfied [12]; and objective list theories, which assess life’s value based on the achievement of prudential goods [13]. However, these theoretical discussions have yet to be fully integrated into empirical medical research on chronic somatic conditions like heart disease.

### Aim

In response to this conceptual and empirical gap, we conducted a study on conceptions of the Good Life of PCHD within the framework of a psycho-cardiological subproject of an interdisciplinary research consortium. The present meta-synthesis constitutes the first part of a two-part investigation aiming to explore which aspects of a good life have already been identified in existing qualitative research involving adults with chronic heart disease. The first step, reported here, examines the emotional significance of chronic heart disease from the perspective of PCHD, drawing on Jaspers’ (1954) concept of *limit situations* – existential experiences such as suffering and death that are inevitable, beyond personal control, yet may prompt reflection on what truly matters in life [14]. Chronic heart disease can be understood as such a limit situation, confronting individuals with their own mortality and the suffering of others [15]. In the second step, we explored people’s ideas of a good life with CHD, described by the literature as important aspects of living well with CHD [16]. This twofold approach was intended to ensure a balanced synthesis of studies that primarily highlight strain and burden, as well as those that emphasize resources and positive adaptation. The central research question guiding this first step of analysis was: How do PCHD experience their disease in terms of a limit situation, and which previously taken-for-granted aspects of life become particularly salient in this context?

## Methods

### Design

This systematic review uses a qualitative meta-synthesis approach with an interpretative focus rather than an aggregative one [17, 18]. To analyze the deep structure of people’s experiences across diverse contexts, Interpretative Phenomenological Analysis [19] (IPA) was used. IPA is grounded in phenomenology, hermeneutics, and idiography. Phenomenology emphasizes the embodied nature of experience, highlighting how chronic disease alters one’s experience, thus providing an appropriate conceptual framework for the present investigation. It considers the lived body (*Leib*) as a subject actively experiencing the world (e.g. Plessner, Merleau-Ponty) rather than the material body (*Körper*), which is usually the object to medical investigations. Hermeneutics (e.g., Gadamer, Heidegger, Ricœur) allows for interpreting latent meanings by relating parts to the whole.

While qualitative analyses usually involve a double hermeneutic process, where researchers interpret participants’ interpretations of their experiences, meta-synthesis adds an additional layer. This “triple hermeneutic” [20] (p.14) underscores the importance of reflecting on the meta-synthesists’ presumptions to ensure the analysis accurately reflects the participants’ experiences rather than researchers’ biases. Given that all authors of this article are investigating the ideas of PCHD of a good life in a qualitative primary study, assumptions were discussed in supervision sessions and efforts were made to manage them following the IPA principle of bracketing. In IPA, bracketing is not understood in the strict Husserlian sense of achieving a presupposition-free stance (epoché), but rather as the active and ongoing process of identifying and temporarily setting aside one’s assumptions to approach the material with openness, before returning to them in reflective engagement. This reflexive stance allows the analyst to challenge preconceptions while hermeneutics provides the means to interpret what is not explicitly stated and to situate it within a broader epistemological context. This process was integrated into the hermeneutic circle of IPA [19] whereby initial readings and interpretations were repeatedly revisited in light of the socio-cultural, temporal, and methodological contexts of the primary studies, thereby realizing IPA’s idiographic focus. Although phenomenological methods for synthesizing qualitative studies exist [21] IPA’s application in meta-synthesis is novel.

Reporting follows the Enhancing Transparency in Reporting the Synthesis of Qualitative Research (ENTREQ) guidelines [22].

### Search strategy

After preregistration on PROSPERO, the study team, consisting of LN and TLB, conducted a systematic literature search in PubMed, PsycInfo, PsycArticles, and PSYNDEX on February 15, 2023. To address the perspectives of PCHD on living with their chronic condition, along with the philosophical concept of the Good Life, we incorporated patient-centered concepts more common in psychology and medicine. Using the medical subject headings (MeSH) “Quality of Life” (QOL) and “Psychological Adaptation” in PubMed, as well as the index terms “Quality of Life” and “Emotional Adjustment” in the psychological databases, we included well-being, life satisfaction, survivorship, coping, PTG, sense of coherence, and salutogenesis in our search. Complete search strings are provided in the Additional File 1.

### Study selection

Following the removal of duplicates, the study team screened 459 articles at the abstract level based on predefined criteria, developed from the PICoS framework, which specified the population, phenomenon of interest, context, and eligible study types [23]. Articles were included if they were written in English or German and reported cross-sectional or longitudinal qualitative studies involving people aged 18 or older with chronic heart disease. To ensure maximum variation, we included studies on different types and severities of heart disease, such as myocardial infarction (MI), cardiac arrest (CA), ischemic heart disease (IHD), heart failure (HF), arrhythmias, and infective diseases. Settings ranged from hospitalization and rehabilitation to advanced home care. Studies were excluded if they focused on congenital heart disease in individuals under 18, severe somatic conditions (e.g., cancer), mental illness (e.g., major depression, schizophrenia), or not addressing the experiences of PCHD. Mixed-methods studies and those involving interviews with people’s relatives or third parties were excluded to maintain a focus on qualitative data and the perspective of PCHD.

**Fig. 1:**
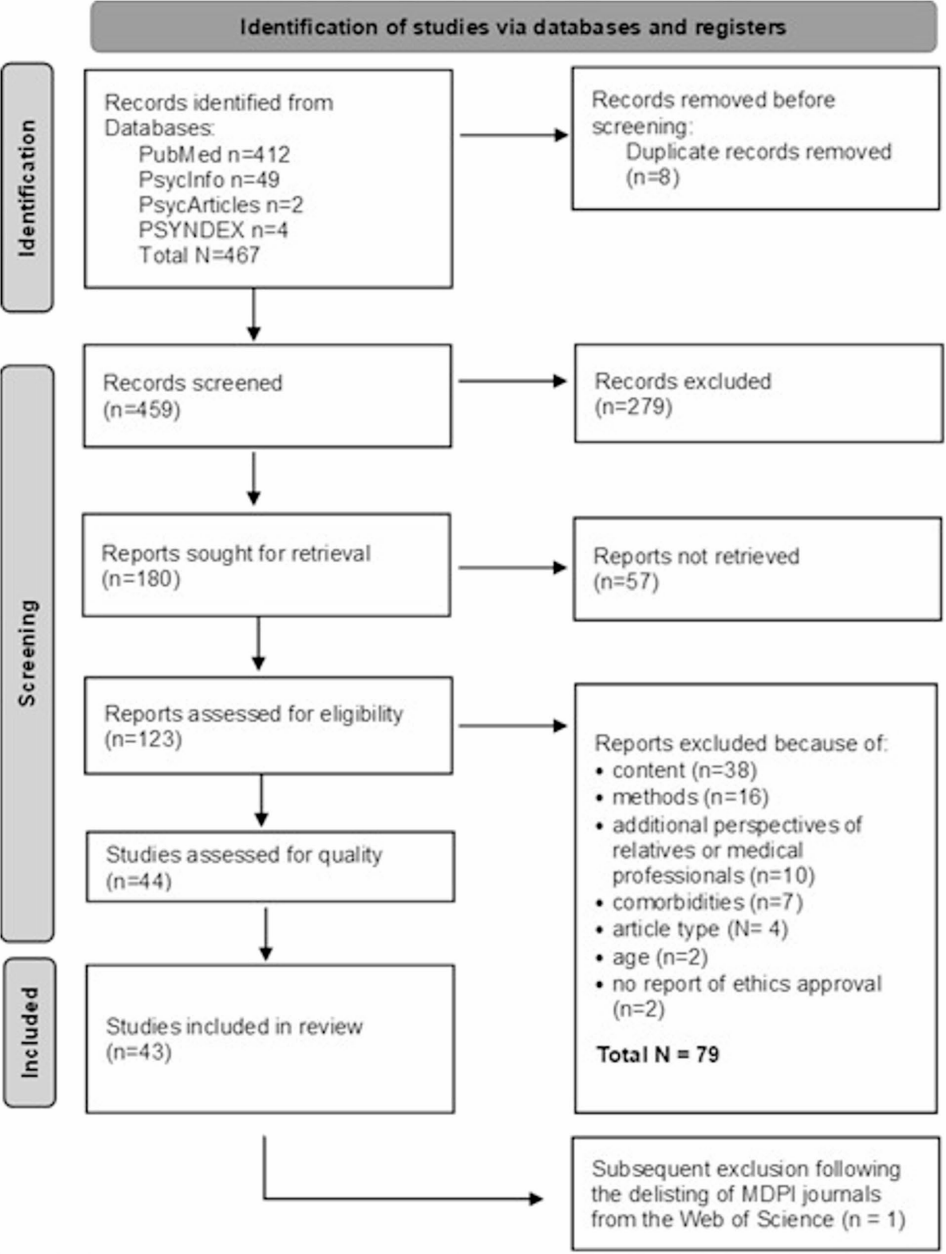
PRIMSA flow chart of the study inclusion and exclusion

After removing 336 articles, the study team reviewed 123 full texts. At this stage, we applied intensity sampling criteria [24] to ensure that included studies provide rich, in-depth descriptions that could be meaningfully interpreted within the philosophical framework of the Good Life. Studies that primarily focused on peripheral aspects (e.g., coping strategies, treatment adherence, or short-term physical recovery following surgical interventions) were excluded, if they did not sufficiently address lived experiences of PCHD from a broader life-course perspective. This process resulted in 44 studies being included in the final synthesis. A follow-up search conducted on 02/15/2024, did not yield additional qualifying articles. The PRISMA flow chart (Fig. 1) illustrates the search process.

### Quality appraisal

In line with intensity sampling, the study team assessed the methodological quality of the 44 included articles using a modified Critical Appraisal Skills Program (CASP) tool, which incorporated the Consolidated Criteria for Reporting Qualitative Research [25] (COREQ) domain on research team and reflexivity to address researcher bias. The appraisal criteria included both COREQ reflexivity and CASP results. To distinguish between limitations in reporting and those related to methodological rigor, we added a “somewhat” category to the standard response options of “yes”, “no” and “can’t tell” [26]. This quality appraisal guided our synthesis by weighing the results of the primary studies: high-quality studies were used to develop initial themes, moderate-quality studies contributed additional codes, and lower-quality studies were utilized to validate the coding system without introducing new codes.

### Evidence synthesis

**Fig. 2:**
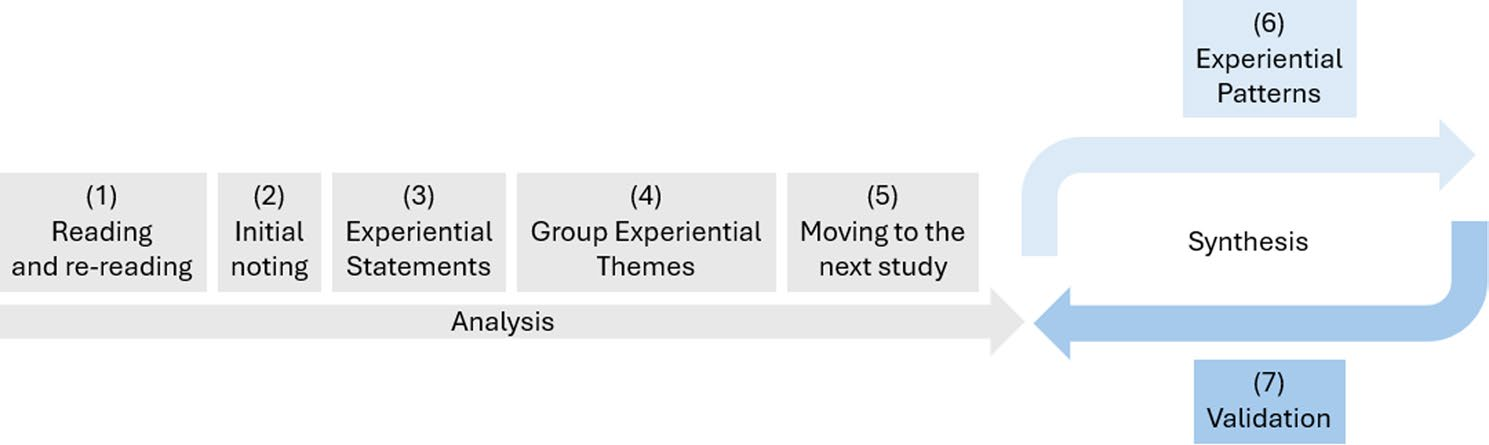
Illustration of the analysis process in terms of IPA, adapted for use in the context of a meta-synthesis

The synthesis followed the steps of IPA, adapted for meta-synthesis, as illustrated in Fig. 2. Steps (1) to (5) represent the analysis of primary study data, where the focus is on understanding and interpreting each individual study. Steps (6) and (7) constitute the synthesis, integrating findings across studies to develop a coherent, overarching understanding of the phenomenon. These steps involve a dialectical approach in the sense of a hermeneutic circle, through which the synthesis is continuously checked against the material and enriched with nuanced aspects. This iterative process is represented by the circle at the right end of Fig. 2.

(1) Reading and re-reading: LN and TLB independently read the first high-quality article in full to develop an initial understanding of the study as a whole. (2) Initial noting: While reading, we noted linguistic and conceptual particularities in first-order data (direct participant quotes), second-order data (contextualizing information), and third-order data (primary authors’ abstract themes). (3) Constructing experiential statements: From first-order data, we developed experiential statements closely aligning participants’ wording. (4) Searching for connections and developing Group Experiential Themes (GETs): Experiential statements were compared, contrasted, and grouped into overarching GETs. (5) Moving to the next study: Steps (1) to (4) were repeated for each subsequent high-quality article. (6) Identifying Experiential Patterns (EPs) across studies: The resulting GETs from all high-quality articles were compiled and examined for recurring EPs. These EPs were then applied to moderate-quality articles and supplemented with additional GETs by returning to steps (3) and (4) where new experiential content emerged. (7) Finally, the EPs were validated against lower-quality articles without generating new codes.

Throughout the process, LN and TLB held weekly meetings to discuss discrepancies, resolve coding differences, and reflect on their own assumptions and positionalities. DB supervised each stage of the study, facilitated resolution of any interpretative discrepancies, and CHL provided additional, occasional support.

The themes presented here emerged from the first step of our analysis, which aimed to develop a deeper understanding of the significance that a chronic heart condition holds for PCHD from a life-course perspective. In the second step of the analysis, which is published elsewhere, we examine ideas of a good life with chronic heart disease of PCHD.

## Results

Based on our deciding criteria (Table 1), we identified 11 high-quality, 17 moderate-quality, and 16 lower-quality articles.

**Table 1:** Appraisal of the included studies with the CASP tool

	High-Quality Articles											Moderate-Quality Articles									Moderate-Quality Articles							Lower-Quality Articles													Lower-Quality Articles		
**CASP / COREQ questions**	1	2	3	4	5	6	7	8	9	10	11	12	13	14	15	16	17	18	19	20	21	22	23	24	25	26	27	28	29	30	31	32	33	34	35	36	37	38	39	40	41	42	43
Clear statement of the studies aim(s)	✓	✓	✓	✓	✓	✓	✓	✓	✓	✓	✓	✓	✓	~	✓	✓	✓	✓	✓	✗	✓	✓	✓	✓	✓	✓	✓	✓	✓	✓	✓	✓	✓	✓	✓	✗	✓	✓	✓	✓	✓	✓	✓
Appropriate qualitative method	✓	✓	✓	✓	✓	✓	✓	✓	✓	✓	✓	✓	✓	✓	✓	✓	✓	✓	✓	✓	✓	✓	✓	✓	✓	✓	✓	✓	✓	✓	✓	✓	?	✓	✓	?	✓	✓	✓	✓	✓	✓	✓
Appropriate design to address the aims of research	✓	✓	✓	✓	✓	✓	✓	✓	✓	✓	✓	✓	✓	?	✓	✓	?	✓	✓	~	~	✓	✓	✓	✓	✓	✓	✓	✓	✓	✓	?	?	~	?	?	✓	✓	✓	✓	✓	?	✓
Recruitment strategy to the aims of research	✓	✓	✓	✓	?	✓	?	✓	✓	✓	~	~	✓	✓	✓	✓	~	?	?	✓	?	?	✓	✓	~	?	✓	~	?	?	✓	?	✓	?	?	?	✓	✓	?	?	?	?	~
Collection of data in a way that addressed the research issue	✓	✓	✓	✓	?	✓	?	~	?	✓	✓	✓	~	?	✓	✓	✓	?	✓	✓	~	✓	?	✓	✓	✓	?	✓	?	✓	?	?	?	✓	?	?	✓	~	?	?	✓	?	✓
**Adequate consideration of the relationship between researcher(s) and participants**	?	✗	✓	✗	✗	✓	✗	✗	✗	~	✗	✗	?	?	✓	✗	✗	✗	~	✗	✗	✗	✗	✗	✗	✗	✗	✗	~	?	✗	✗	✗	?	✗	✗	✗	✗	✗	✗	✗	✗	~
**Personal characteristics of the research team (COREQ)**	✓	✓	✓	✓	✓	✓	✓	✓	✓	✓	✓	✗	~	~	✓	~	✓	✗	~	✗	✗	✗	✗	✓	✗	~	✗	✗	~	~	✗	✗	~	~	✗	~	✗	~	✗	✓	✗	✗	~
Ethical issues considered	✓	✓	✓	✓	✓	✓	✓	~	✓	✓	~	✓	✓	~	✓	~	✓	~	~	~	~	~	~	✓	✓	✓	✓	?	✓	✓	✓	?	~	~	~	✓	~	✓	~	~	✓	~	~
**Sufficient and rigorous data analysis**	✓	✓	✓	✓	✓	✓	✓	✓	✓	✓	✓	✓	✓	✓	~	✓	~	✓	✓	✓	✓	✓	✓	~	✓	✓	✓	~	?	~	?	~	?	~	?	?	?	~	?	~	~	~	~
**Clear statement of findings**	✓	✓	✓	✓	✓	~	~	~	✓	~	~	✓	~	✓	~	✓	✓	✓	✓	✓	✓	✓	✓	✓	✓	~	✓	~	~	✓	✓	~	✓	✓	✓	✓	✓	✓	~	✓	~	✓	✓
Research valuability	✓	✓	✓	✓	✓	✓	✓	✓	~	✓	~	~	✓	✓	✓	✓	~	✓	✓	✓	✓	✓	✓	✓	✓	✓	✓	✓	✓	✓	✓	~	✓	✓	?	✓	✓	✓	~	~	~	~	✓

*✓* means ‘yes’, ✗ ‘no’, ? ‘can’t tell’ and ~ ‘somewhat’. The numbering corresponds to that in Supplementary Material 2. Bold rows indicate the deciding criteria

One lower-quality article was subsequently excluded because the publishing MDPI journal was delisted from the Web of Science in 2024 [27], resulting in a final sample of 43 studies with a total of 753 individuals, of whom 294 were female and 459 were male. Two lower-quality studies did not report exact gender distribution. Age ranged from 18 to 99 years, with means and medians between 40 and 79 years. The duration of illness varied from 3 months to 25 years. As chronic heart diseases often co-occur, several studies included samples with more than one type of cardiac condition. Most studies were conducted in Scandinavian countries, with 18 of the 27 European studies originating there. Six of the Asian studies included samples from the Middle East, and one study each from Taiwan and Singapore. Six studies originated in the United States, and two in Canada. Table 2 provides an overview of the sample characteristics. Detailed study characteristics are displayed in Additional File 2.

**Table 2:** Sample characteristics

Characteristic	Total (%)	Characteristic	Total (%)
Male gender	459 (66)	Method of analysis	
Studied type of heart disease		Phenomenology	19 (44)
Cardiac Arrest	7 (16)	Content Analysis	16 (37)
Myocardial Infarction	11 (23)	Grounded Theory	8 (19)
Coronary Heart Disease	6 (14)	Text Condensation	1 (2)
Heart Failure	15 (35)	Geographical region	
Arrhythmia	4 (9)	Europe	27 (63)
Infective Endocarditis	1 (2)	Asia	8 (19)
Heart Valve Disease	2 (4)	North America	8 (19)

Our analysis indicates that PCHD perceive their heart disease as a major disruption in their life trajectory, creating a pronounced contrast between life before and after the onset of illness. This experience is encapsulated in the overarching theme of “The broken flow of life,” which is further detailed through the subthemes: “I no longer feel safe in my body,” “Suddenly, I have less of a future,” “My identity feels shattered,” and “My disease strains my relationships.” The following sections provide an in-depth discussion of these themes.

### The broken flow of life

The overarching theme emerged from all the articles included, summarizing people’s experience of their heart disease as a biographical rupture. Their altered physical condition leads to a break with life expectations that PCHD had implicitly assumed based on their prior state of health. A 55-year-old man, who has been living with a defibrillator for several decades due to his HF, describes the rupture with his life expectations at the time of implantation particularly vividly:

“My plan was to…get married, and do what everybody else does. What happened to me is [I’m] in [my] 20’s…and all of a sudden…they’ve got to put a box [defibrillator] in [me]…And that kind of changes things…I mean, I’m just trying to stay alive, and so dating is gone…The girl I was dating at the time, God I feel sorry for her [because] she never heard from me again…I wasn’t about to get her involved in that….” [28] (p.3).

The acute threat brings the preservation of one’s life to the forefront, interrupting the life course typically shared with peers and implicitly perceived as “normal.” Heart disease leads to significant alterations in people’s bodily experiences, relationships, and self-images. The future, once envisioned through the lens of their lived past, now appears diminished in both scope and richness. This perceived disconnect between their past, present, and altered future perspectives might in some cases contribute to a loss of meaning in life.

### I no longer feel safe in my body

Experiencing one’s own body as a threat to life is reported in all included studies on arrhythmia and in nearly all studies involving survivors of MI and CA, as well as in people with IHD and HF. The loss of a previously taken-for-granted sense of bodily safety is interconnected with the experience of vulnerability to death, a sense of alienation from one’s own body, and the perceived inability to manage daily activities due to reduced stamina and fatigue.

Most of the included studies examine people’s experiences of acute cardiac events, which expose them to vulnerability and mortality. Confronted with these realities, PCHD live with the constant fear of experiencing another cardiac event that could lead to sudden death. In studies on HF, people report both the fear of unpredictable deterioration in their health status, particularly of “suffer[ing] in the end” [29] (p.99), as well as the fear of sudden symptom attacks. The abrupt onset of a life-threatening situation is described as distressing experience:

“It is a matter of seconds; I mean a heart attack only takes a few seconds” [30] (p.232).

The constant fear of an imminent and unpredictable life-threatening event forces PCHD to remain in a perpetual state of preparedness for the worst-case scenario. A woman with arrhythmia clearly demonstrates how the loss of bodily safety profoundly impacts her daily life:

“I have to drive for work, but I’m always scared it might happen while I’m driving. Now I always drive in the right hand lane, so if it happens, I can pull over to the shoulder quickly.” [31] (p.6).

To timely recognize a sudden emergency, PCHD report a heightened awareness of bodily processes. However, PCHD often find it challenging to distinguish between signs of severe events and normal bodily processes. This difficulty seems to be particularly distressing when PCHD have previously experienced dismissal of their initial symptoms by healthcare professionals or relatives. Moreover, it is exacerbated after hospital discharge, when PCHD no longer have professional support to manage bodily reactions, leading to increased feelings of isolation and heightened bodily fears. A MI survivor described how persistent physical changes continuously challenged the feeling of bodily safety and exacerbated the experience of existential fear:

“I was very tired and often felt stabbing chest pains. I would keep silent, terrified, thinking: ‘Shall I die? Is that MI again?’ I could not even move my body.” [32] (p.39).

Regardless of the type of heart disease, PCHD lose a natural sense of trust in their bodies, which they begin to perceive as unreliable and threatening. While devices and medications can help PCHD rebuild this trust by offering a sense of protection against sudden cardiac events, they also introduce their own challenges. PCHD report experiencing pain and feeling restricted by the device, which, although it may alleviate the fear of sudden death, can also generate anxiety about potential shocks, and heighten awareness of their risk of dying.

### Suddenly, I have less of a future

The experience of an unreliable and restricted body leads PCHD to anticipate a future that is both uncertain and limited in quantity and quality. Across studies, the fear of sudden or premature cardiac death and the unpredictability of their condition shaped participants’ perceptions of what the future could hold. Slightly more than half of the studies involving people with HF report a changed perception of the future, while 44% of studies on MI, and two out of six studies on IHD noted similar findings. Among studies examining CA survivors’ experiences, only those two focusing on people living with implantable Cardioverter Defibrillator (ICD) report an altered outlook. The predominant theme across all studies was the fear of sudden and premature cardiac death.

Some participants explicitly described uncertainty regarding their longevity, as illustrated by a woman at least two years post-MI:

“Life is not, it is not something obvious anymore, I don’t know if I will live tomorrow, or not.” [30] (p.232).

For others, this uncertainty led to the perception that planning and goal-setting were futile, increasingly shifting their attention to the present. A survivor of an out-of-hospital cardiac arrest (OHCA) described this dynamic:

“I live more in today than that I think of the future. I try to, but you often encounter things that make you think ‘Why am I doing this?’ I have taken up an old hobby and am in the process of putting together a new collection for next year. Then you get to the point where you think ‘you’re doing that, but will you be alive then?’” [33] (p.1013).

Interpreting this description of uncertainty reveals an additional experiential dimension: uncertainty about the future appears to translate into a sense of limitation. When the future is uncertain, people may perceive stronger limitations on what is achievable or meaningful in their lives, affecting both quantitative aspects (such as lifespan or life opportunities) and qualitative aspects (such as personal goals and meaningful experiences).

Both people with HF and MI report feelings of hopelessness and discouragement due to a lack of prospects for improvement or the inability to return to their previous lives. Persistent or recurring restrictions result in a future that is constrained not only in quantity but also in quality. People with HF and IHD report feeling restricted in their ability to shape their futures. This is especially true for younger PCHD, for whom chronic heart conditions can significantly hinder career aspirations and family planning. A young woman with HF illustrated how medical limitations imposed permanent changes on her life trajectory:

“Because my status was very serious when I was first diagnosed, my husband and I… were advised to no longer… try to become pregnant. My heart was too weak to go through childbirth. I was sad, but I understood. It wasn’t fair to bring a child into the world with… such a very sick mommy…. Being a childless mother is a fallout of my CHF.” [28] (p.98).

Confronted with an uncertain future that may diverge from their desires for a fulfilled life, people with HF as well as CA and MI survivors report an increased focus on the present. While hope for the future is vital for well-being, it underscores the gap between reality and personal aspirations, as illustrated by a middle-aged person with moderate to severe chronic HF:

“One cannot plan so much//But, of course, one hopes for…things to be the way they used to be, that one will…feel good. …That is important…But…no, I don’t plan much…. Well, sometimes one gets carried away…but then one realizes, it won’t work…//Then one realizes one is limited” [34] (p.8).

Thus, chronic heart conditions impose both quantitative and qualitative limitations on participants’ futures, shaping experiences of meaning, life planning, and goal pursuit. Our interpretation highlights that these limitations are not only a consequence of uncertain longevity but also emerge from the lived experience of what is realistically achievable, illustrating the intertwining of *uncertain* and *limited future* in PCHD.

### My identity feels shattered

The experience of being deeply confounded in identity is the most prominent theme across all types of heart disease. Each of the included studies on arrhythmia and IE and at least two thirds of the studies including people with HF and IHD, as well as MI and CA survivors identify this issue.

Physical changes that often necessitate lifestyle adjustments highlight for PCHD the break with their previous self-image. Consequently, PCHD often feel unable to actively shape their lives in alignment with their previous self-image. For example, a woman who “was physically active, both in her leisure time, at the house, at work, and with sports” [35] (p.1449) before experiencing an OHCA describes:

“I do not feel self-control, only frustration. It is hard to acknowledge that nothing will be as before. I have felt like Alice in Wonderland who enters this totally new world. Who am I now and what is this? I do not know whether to go left or right or which door to open. It has been and is a huge work for me to find out who I am and what am I going to do.” [35] (p.1449).

By likening her situation to that of Alice in Wonderland, this woman underscores her sense of alienation from her previous position in life. She describes feeling profoundly disoriented in her identity, which impairs her ability to make autonomous decisions about her future.

The disruptions in self-image most reported by PCHD relate to their perceived age, activity and productivity, as well as the independence and self-determination they have experienced thus far.

“I’m 54 years old – still young – and when I see 70 year olds walking the street and passing me by I feel like a right ejit (idiot) – they’re flying up the street and I’m crawling up it” [36] (p.227).

By contrasting their own cumbrous movements with the light-footedness of much older individuals, this person with advanced HF underscores the profound impact of the disease on the perceived age. For younger PCHD in particular, the disease represents a stark rupture with their previous image of an active self, as described by a middle-aged person with IE:

“Now I have small children, I can’t do a lot of things with them anymore. So for me, it has been a major change from doing sports every day and going to work. I haven’t been able to do that.” [37] (p.14).

The loss of occupational engagement is discussed in 10 studies and is linked to various other losses, such as diminished self-esteem, a lack of fulfillment and purpose, decreased social interactions, and financial strain. This experience seems particularly striking for men, with three studies noting that male participants felt a disruption in their perceived role as providers, leading to feelings of insufficiency, and worthlessness. In contrast, women reported feeling inadequate in their role as mothers and guilty for drawing attention due to their heart condition. Additionally, they faced the stereotype that women do not develop heart disease.

Job loss may result in financial hardship and dependency, contributing to a broader perceived loss of autonomy reported in 15 studies. Many PCHD view increased reliance on medical devices, medications, and social support as significant threats to their autonomy and self-worth. This sentiment is exemplified by an 82-year-old man who, several months after surviving a MI, expressed:

“…I’m used to doing everything myself. You get overlooked – like you feel worthless” [38] (p.371).

As reflected in this man’s interpretation of others’ behavior, the experience of identity incoherence also affects the relational experiences of PCHD, which is explored in the following theme.

### My disease strains my relationships

All the included studies on IE and arrhythmia, and at least two-thirds on CA, MI, and HF, respectively, report strained relationships. However, only half of the studies on the experiences of people with IHD address this theme. Key dynamics identified include feelings of abandonment and isolation, being a burden to others, and experiencing overprotection from others. While the feeling of becoming a burden on others is common across cardiac conditions, abandonment and isolation are particularly prevalent among people with HF. Especially those with reduced mobility due to severe HF often rely on others for social integration. For instance, an older person in palliative home care with New York Health Association (NYHA) stage III-IV HF explained:

“When you’re ill you’re not interesting anymore. Before I used to go visiting friends and they used to visit me and that was nice and fun but these days hardly anyone drops by.” [39] (p.299).

According to this person, the loss of mobility and activity is closely linked to how interesting one’s company is perceived by others. However, feelings of abandonment stem not only from reduced mobility but also from a lack of acknowledgment of symptom burden, leading to a sense of not being understood, a sentiment expressed by people across all types of heart diseases. One CA survivor who received an ICD within the year prior to the interview shared the emotional burden of not feeling understood while living with an invisible chronic disease:

“I sometimes feel that people don’t understand. At first sight you look fine, if I may say so, because you’re basking in good health. And yet you have limitations. And I think that they don’t understand that very well. Someone who’s in a wheelchair doesn’t have to say that he isn’t well all the time. And if they say, when I meet them, ‘how are you?’ and I say ‘well, could be better’, then they say ‘but you look fine!’” [31] (p.1013).

Another significant issue for CA survivors, compared to those with other heart conditions, was the feeling of not being taken seriously by healthcare professionals, leading to a lack of necessary support. This involved psychological assistance for coping with emotional challenges, particularly the fear of dying. By exposing their families to a potentially traumatic event and typically taking on caregiving responsibilities despite their own emotional distress, survivors of acute cardiac events often perceive themselves as a burden to their relatives. To prevent adding strain on their already anxious relatives, some PCHD conceal their symptoms or avoid discussing emotional issues. A father who survived an OHCA stated,

“The worst thing is that you wake up to a traumatised wife and kids. My biggest challenge has been to relate to my family before I have been able to relate to myself” [35] (p.1448).

Parents are particularly concerned about the psychological and physical impacts of their disease on their children, fearing how potentially traumatic experiences may affect them and whether they can cope with necessary lifestyle changes. While worried about the heritability of their condition, they notice their children feeling responsible for their recovery and well-being.

Although PCHD appreciate the support they receive, they often find ongoing protective behaviors from others undermining their autonomy, leading to discomfort with unsolicited advice and excessive concern.

“After the MI, the family started to look at me a little differently…like I am sick, not as a healthy person […] I simply do not like it! I would rather be seen as a normal healthy person, it doesn’t suit me to be looked at as a patient!” [32] (p.39).

This person describes how the worried looks of family members reinforces his identity as a patient. Although intended as support, overprotective behavior increases the awareness of PCHD of their illness, contributing to their sense of being different from others and from their former selves, which exacerbates the experience of heart disease as a disruption of their normal life.

### Interpretation and discussion

This meta-synthesis of 43 qualitative studies explores how adult PCHD experience their condition, emphasizing not only symptom burden, functional limitations, and well-being but also the broader existential, relational, and temporal dimensions of a Good Life. While previous meta-syntheses have primarily focused on barriers and facilitators to treatment and health behaviors [40–44]to our knowledge, this is the first meta-synthesis to frame the lived experiences of PCHD within the philosophical concept of the Good Life, which allows us to consider how CHD disrupts the people's sense of normalcy, bodily integrity, identity, and future orientation. This approach highlights aspects of lived experience that conventional HRQOL assessments often overlook. In the following, our findings are integrated into existing evidence and interpreted with regard to their implications for psychological and medical treatment.

### Lost sense of bodily trust

The physical dimension of HRQOL typically assesses functional restrictions due to symptoms. In line with previous meta-syntheses of experiences among CA [45] and MI [46] survivors, as well as people with ICD [47, 48] and HF [49–51] we identified the more complex experience of physical insecurity, associated with a heightened awareness of vulnerability, and fears of ICD shocks, hospitalization, and death. For people with HF, however, we also identified concerns about ongoing health deterioration – an issue inconsistently reported in the literature. While one meta-synthesis describes the experience of HF as a progressively worsening condition [49] another suggests that it is perceived as episodic rather than chronic [50]. The latter also emphasizes a disconnect between people’s subjective bodily experiences and physicians’ symptom explanations, exacerbating feelings of bodily insecurity. This underscores the importance of healthcare providers using language that reflects the subjective experiential qualities of PCHD, rather than focusing solely on symptom descriptions. To better understand bodily and self-experience beyond symptom and ability clusters, phenomenologically inspired theories are particularly valuable. Carel’s concept of bodily doubt encapsulates a loss of continuity, transparency, and faith in one’s body [52] drawing on Ratcliffe’s phenomenological analysis of existential feelings, tacit knowledge underlying our perceptions of the world, that become evident when disrupted [53]. Persistent fear of death increases awareness of bodily reactions, described as interoceptive hypervigilance [54]. The body, no longer functioning as it once did, feels alien, fostering bodily doubt and existential uncertainty [54, 55]. According to Maslow’s hierarchy of needs, safety is a fundamental requirement that must be fulfilled before self-actualization is possible [56]. Its deprivation can worsen illness and hinder personal growth, making a sense of bodily safety crucial for a fulfilling life.

### An altered future perspective

The perceived uncertainty of survival also affects the temporal experience of PCHD. A future that is perceived as uncertain and therefore shrinking in both length and richness hinders PCHD from making plans for their future and deprives them of a sense of meaning. A changed perception of the future is explicitly reported in meta-syntheses on the experiences of people with HF [45] and CA survivors [49]. However, this alteration of temporal experience should not be confused with project-specific hopelessness regarding the achievement of certain goals. Rather, it is the loss of hope for a fulfilling and self-determined future that is deeply embodied and can be understood as a disruption of fundamental trust, akin to demoralization [53]. A large study involving 12,895 U.S. men and women identified vital exhaustion, which includes the element of demoralization, as a significant risk predictor for cardiac events [57]. Therefore, we conclude that the altered experience of the future should be considered when assessing HRQOL.

### Lost sense of self

Another aspect of altered temporal experience is reflected in the people’s confounded sense of self. According to Fuchs [58], the sense of identity is highly embodied – personal identity is shaped by engaging with the environment in a routine manner. PCHD who can no longer live according to their previous attributes of activity, autonomy, and self-determination experience a profound disruption of their identity.

Our findings suggest that especially younger PCHD perceive their condition as a disruption of the natural life course as their symptoms limit life planning and daily functioning. This is consistent with age-related effects on QOL and psychopathology in chronic heart disease [59–62]. The inability to pursue life-phase-specific goals or meet age-related social role expectations highlights the rupture between past and future. In line with previous findings, we identified gender differences in terms of social roles [46, 47]. While men expressed concerns about their role as providers and feared financial difficulties, women report being concerned about not being able to fulfill their maternal roles. Additionally, women faced the stereotype of not being expected to have heart disease – an experience leading to the perception of “having a man’s illness in a man’s world" [46] (p. 414). With heightened awareness for gender-sensitive medicine, the number of studies examining the impact of sex on risk factors for cardiovascular disease, medical outcomes, and psychosocial variables has increased [63]. However, the question of whether gender-specific concepts of a good life exist and influence living with chronic heart disease remains an underrepresented research topic.

### Burdened relationships

Our synthesis identified four conflict themes that lead PCHD to perceive their relationships as strained. These include feeling abandoned and misunderstood by others, while perceived overprotection evokes feelings of being a burden to others. These issues are also reported in previous meta-syntheses [47, 48] and align with a phenomenological hermeneutic investigation of the meaning of support in 5 elderly women with HF. This study indicates that a lack of support is perceived as abandonment, resulting in feelings of loneliness, whereas supportive relationships promote independence without causing feelings of being a burden [64]. Interestingly, in our analysis, the experience of loneliness and isolation emerged as a theme predominantly reported in studies on HF. However, a systematic investigation of heart disease-specific relationship dynamics is necessary to make further conclusions.

### Strengths and limitations

To our knowledge, this is the first meta-synthesis to examine the experiences of PCHD through the lens of the philosophical concept of the Good Life, enabling consideration of broader aspects and temporal dimensions of these subjective experiences. The novel application of IPA to synthesize primary research data allowed us to develop themes that reflect the core structure of people’s perspectives on their chronic conditions. Unlike previous meta-syntheses, we included a range of cardiac conditions to reflect the realistic diversity of the heterogenous group, thereby revealing distinct relational stressors across subgroups of PCHD.

A methodological strength is the explicit incorporation of reflexivity criteria into our quality appraisal process. While we used the CASP checklist for its high degree of comparability and transferability across qualitative reviews [26], we modified it by adding items from the COREQ domain on research team and reflexivity, ensuring that authors not only reported engaging in reflective processes, but also provided information on interviewer identity, occupation, experience, gender, and credentials. We also implemented a fourth rating category (“somewhat”) to differentiate between partial fulfillment of a criterion and complete absence, thus addressing the challenge of distinguishing between issues of conducting and reporting research, which has already been highlighted [26]. We acknowledge that other critical appraisal tools, such as the framework proposed by Popay et al. (1998) [65] with its emphasis on privileging lay accounts and subjective meaning, might have been particularly aligned with our research aim of exploring people’s lived experiences. Compared to such more flexible approaches, CASP provides less room for interpretative discretion, which may have led to a more conservative rating of included studies. Future meta-research in this field could benefit from combining the structured clarity of CASP with the ontological sensitivity of Popay et al.’s criteria, to better capture the complexity of qualitative evidence on lived experience.

Further limitations must be noted: the varying number of studies across different heart disease types limits interpretation of subgroup differences, underscoring the need for further systematic research. The impact of other chronic conditions or aging on people’s perceptions of their condition remains unclear. Missing data on key factors such as age, gender, disease duration, and severity limited the application of IPA’s idiographic approach and prevented full representation of experiential diversity. It is worth noting that, while IPA thrives on an idiographic orientation, this potential can only be fully realized in meta-synthesis when the specific socio-cultural, temporal, and methodological contexts of primary studies are given particular weight. In our case, the large number of included studies, the diversity of variables potentially influencing the phenomenon under investigation, and the breadth of the phenomenon itself restricted our ability to capture these contextual nuances. Finally, most included studies originated from Western, Educated, Industrialized, Rich, and Democratic (WEIRD) contexts. These limitations should be considered when interpreting our findings, which nonetheless offer important insights into the lived experience of PCHD and may inform more patient-centered approaches to care and research design.

## Conclusion

By synthesizing the lived experiences of PCHD within the framework of the Good Life, this meta-synthesis highlights HRQOL dimensions that extend beyond symptom burden, functional limitations, and well-being. Existential, relational, and temporal aspects, such as bodily doubt, biographical rupture, and altered future perspectives, emerge as central to understanding the impact of CHD on people’s lives. Integrating these dimensions into HRQOL assessments could foster more patient-centered care and better support patients in achieving a meaningful and fulfilling life despite chronic illness.

## Supplementary Information

Supplementary Material 1.

Supplementary Material 2.

Supplementary Material 3.

## Data Availability

The datasets supporting the conclusions of this article are included within the article and its additional files.

## Abbreviations

PCHD: People with Chronic Heart Disease
HRQOL: Health-Related Quality of Life
ENTREQ: Enhancing Transparency in Reporting the Synthesis of Qualitative Research
PROSPERO: International Prospective Register of Systematic Reviews
CASP: Critical Appraisal Skills Program
PRISMA: Preferred Reporting Items for Systematic Reviews and Meta-Analyses
QOL: Quality of Life
PTG: Posttraumatic Growth
IPA: Interpretative Phenomenological Analysis
MeSH: Medical Subject Headings
MI: Myocardial Infarction
CA: Cardiac Arrest
IHD: Ischemic Heart Disease
HF: Heart Failure
COREQ: Consolidated Criteria for Reporting Qualitative Research
ICD: Implantable Cardioverter Defibrillator
OHCA: Out-of-Hospital Cardiac Arrest
NYHA: New York Health Association
