# Supplementary material for: “That kind of changes things”: a meta-synthesis of the lived experiences of people with chronic heart disease
Source: Health Qual Life Outcomes. 2025 Sep 18;23:84. doi: 10.1186/s12955-025-02423-6 (PMC12447608; doi:10.1186/s12955-025-02423-6)
Supplement: Supplementary file 2 — Supplementary Material 2. [file 12955_2025_2423_MOESM2_ESM.docx]

**Table 2.** Basic features of the included studies in the meta-synthesis

| Study, Year, Country | Aim/ Phenomenon under investigation | Methodology and methods | Major findings (as presented by primary authors) |
| --- | --- | --- | --- |
| 1  Sert et al. 2021,  Turkey | To examine the effect of Implantable Cardioverter-Defibrillators (ICD) on people’s lives and their experiences of worrying about death | Sample:   - 26 people living with ICD - living with ICD for 2-108 months, $\bar{x}$=47 months - 7 women, 19 men - aged 30-77 years, $\bar{x}$=58 - comorbidities: hypertension, HF   Data collection:  Semi-structured interviews  Data analysis:  Giorgi's Descriptive Phenomenology method | 3 themes and 10 subthemes:   1. Physical impact of the device on the body    1. Changes in physical functioning    2. Like part of the body 2. Social impact in daily life and adaptation to restrictions    1. Necessary for life    2. Restrictive behaviors of day-to-day life    3. Seeking social support 3. Emotional impact of the experience of shock and worry about death    1. Inevitable truth    2. Living at the boundary    3. Every shock is like you are going to die    4. Advancing on an uncertain path    5. Trust in life insurance |
| 2  Wagner et al. 2021,  Denmark | To gain in-depth understanding of how Out-of-Hospital Cardiac Arrest (OHCA) survivors experience short- and long-term consequences in daily life | Sample:   - 33 OHCA survivors - 8 women, 25 men - aged 40-83 years, $\bar{x}$=59 - $\bar{x}$=16 months since OHCA   Data collection:  Focus group with 4-7 participants  Data analysis:  Ricœur’s Phenomenological Hermeneutics | 3 narratives:   1. A fragmented memory at the mercy of the system 2. Living in the shadow of anxiety and mixed feelings 3. Lost sense of self |

**Table 2.** Continued

| Study, Year, Country | Aim/ Phenomenon under investigation | Methodology and methods | Major findings (as presented by primary authors) |
| --- | --- | --- | --- |
| 3  Nordfonn et al. 2020,  Norway | To explore how people with Heart Failure (HF) perceive their capacity to manage treatment and self-care | Sample:   - 17 people with HF - 6 women, 11 men - aged 46-74 years, $\bar{x}$=62 years - HF diagnosis at least 3 months prior to the interview   Data collection:  Semi-structured interviews  Data analysis:  Systematic Text Condensation (STC) | 3 themes and 8 Subthemes:   1. Personal Characteristics    1. Inherent strength    2. Maintenance of a positive attitude 2. Coping strategies    1. Selective denial    2. Ability to adapt by setting new goals    3. Careful selection of information 3. Emotional and informative support    1. Support from health care professionals enhancing patient capacity    2. Support from next to kin in patient’s self-care    3. Practical support and hope from peers |
| 4  Falk et al. 2006,  Sweden | To describe how people living with Chronic Heart Failure (CHF) perceive the maintenance of their daily life | Sample:   - 17 people with CHF - 5 women, 12 men - aged 55-83 years, $\bar{x}$=72 years - 1-12 years of illness - 11 participants with NYHA class III, 6 participants with NYHA class IV   Data collection:  Semi-structured interviews  Data analysis:  Phenomenographic (based on Gothenburg University approach) | 5 categories and 14 subcategories:   1. Dealing with the realities of life 2. To adjust activities 3. To work in collaboration 4. To use creativity 5. Dealing with thoughts about life's infinity 6. To live with thoughts — past, present and future 7. To live with the state of mind 8. Taking responsibility 9. To follow treatment regime 10. To make one's own decisions 11. To use one's knowledge 12. Dealing with the surrounding world 13. To experience continuity 14. To feel trust 15. To be part of a social network 16. To deal with the local environment 17. Keeping up with values of life: 18. To retain spare time activities 19. To retain lifestyle |

**Table 2.** Continued

| Study, Year, Country | Aim/ Phenomenon under investigation | Methodology and methods | Major findings (as presented by primary authors) |
| --- | --- | --- | --- |
| 5  Kristofferzon et al. 2007, Sweden | The experiences of everyday life of women and men 4–6 months after Myocardial Infarction (MI) and their expectations for the future | Sample:   - 39 people with MI - 20 women, 19 men - aged 47-90 years - Interviews took place at least 1 month after MI   Data collection:  Semi-structured interviews  Data analysis:  Graneheim and Lundman’s qualitative content analysis | 2 themes and 8 categories:   1. Managing consequences of MI 2. Health problems 3. Lifestyle modifications 4. Emotional reactions 5. Work and social activities 6. Professional and lay network 7. Finding a meaning in what has happened 8. Positive consequences of MI 9. Life values 10. Hopes |
| 6  Rasmussen et al. 2015, Denmark | People’s experiences of recovery after Infective Endocarditis (IE) | Sample:   - 11 people with IE - 3 women, 8 men - aged 29-86 years, $\bar{x}$= 63 years - Interviews took place 3 to 6 months after treatment   Data collection:  Semi-structured interviews  Data analysis:  Ricœur’s phenomenological hermeneutics | 3 themes:   1. An altered Life 2. Shocking Weakness 3. Road to recovery |

**Table 2.** Continued

| Study, Year, Country | Aim/ Phenomenon under investigation | Methodology and methods | Major findings (as presented by primary authors) |
| --- | --- | --- | --- |
| 7  Johansson et al. 2003, Sweden | Women’s experiences following MI | Sample:   - 8 women with MI - 2-25 years after MI - age is not reported   Data collection:  Semi-structured interviews  Data analysis:  Based on Giorgi's descriptive phenomenology method | 5 themes:   1. My heart and my life 2. Living with an unreliable body 3. When life fails 4. Feeling vulnerable and lacking participation 5. Trying to achieve reconciliation |
| 8  Bremer et al. 2009, Sweden | Describing people’s experiences of surviving OHCA | Sample:   - 9 OHCA survivors - 1 woman, 8 men - aged 44-70 years - Interviews took place 6 months to 15 years after discharge   Data collection:  Semi-structured interviews  Data analysis:  Hermeneutic phenomenological approach (Dahlberg 2008; Gadamer 1995, 1960) | 7 themes:   1. Sudden and elusive threat 2. Awakening in perplexity 3. The memory gap: A loss of coherence 4. Searching for coherence 5. Distressing and joyful understanding 6. Existential insecurity exposed by feelings of vulnerability 7. Well-Being through coherence and meaning in life |
| 9  Nadarajah et al. 2016, USA | Recovery experiences of people with a predominantly positive outlook after acute cardiac event | Sample:   - 10 CA survivors in cardiac rehabilitation with a positive outlook - 4 women, 6 men - aged 46-75 years   Data collection:  Semi-structured interviews  Data analysis:  Colaizzi’s phenomenological method | 3 themes:   1. Choosing life over death 2. Learning to live a new self 3. A life-transforming cardiac event |

**Table 2.** Continued

| Study, Year, Country | Aim/ Phenomenon under investigation | Methodology and methods | Major findings (as presented by primary authors) |
| --- | --- | --- | --- |
| 10  Nordgren et al. 2007, Sweden | Living with moderate to severe CHF as a middle-aged person | Sample:   - 7 people with CHF - 3 women, 4 men - aged 38-66 years - NYHA class III- IV - hospitalization in previous year   Data collection:  Semi-structured interviews  Data analysis:  Phenomenological lifeworld perspective (based on Dahlberg and Giorgi) | 3 constituents:   1. An ambiguity of the body 2. Losing track of life 3. Balancing life |
| 11  Allen et al. 2009,  USA | This study sought to better understand the lived experience of women with NYHA class III HF | Sample:   - 4 women with HF - aged 49-64 years - comorbidities: anemia, depression, coronary artery disease   Data collection:  Narrative (Single Question) interviews  Data analysis:  Based on Giorgi's Descriptive Phenomenology method | 5 themes:   1. Developing a new conception of self 2. Conceding physical limitations 3. Enduring emotional heartache 4. Accepting support 5. Rejuvenating through rest |

**Table 2.** Continued

| Study, Year, Country | Aim/ Phenomenon under investigation | Methodology and methods | Major findings (as presented by primary authors) |
| --- | --- | --- | --- |
| 12  Moshki et al. 2019,  Iran | This qualitative study sought to scrutinize the experiences of people with HF and the perceived positive effects of living with the disease | Sample:   - 19 people with HF - 6 women, 13 men - aged 37-86 years - disease duration: $\bar{x}$=5.2 ± 2.2 years   Data collection:  Semi-structured interviews  Data analysis:  Framework Analysis (based on Ritchie and Spence) | 6 themes and 14 subthemes:   1. Healthy Lifestyle 2. Changes in nutritional status 3. Behavioral Change 4. Effective Interactions 5. Feeling close to family 6. New relationships 7. Appreciation of life 8. Appreciation of everyday life 9. Appreciation through social comparisons 10. Spirituality 11. Finding meaning 12. An Increase in faith and closeness to God 13. Optimism 14. Reappraisal of life and priorities 15. Adoption of new decisions 16. Adherence to morality 17. Endurance 18. Becoming stronger 19. Gaining self confidence 20. Role modelling |
| 13  Bremer et al. 2019, Sweden | describing people’s experiences of surviving OHCA | Sample:   - 9 OHCA survivors - 1 woman, 8 men - aged 44-70 years - Interviews took place 6 months to 15 years after CA   Data collection:  Semi-structured interviews  Data analysis:  Phenomenological Reflective Lifeworld research (based on Dahlberg) | 2 themes with 2 subthemes each:   1. Striving to live in everyday life    1. Struggling to reach a new identity    2. Searching for existential wholeness in a fragmented world 2. Striving for security    1. Vulnerability during transition to home    2. Abandonment at home |

**Table 2.** Continued

| Study, Year, Country | Aim/ Phenomenon under investigation | Methodology and methods | Major findings (as presented by primary authors) |
| --- | --- | --- | --- |
| 14  Seah et al. 2016,  Singapore | the experiences, needs, and coping strategies of people living with HF in Singapore | Sample:   - 15 people with HF - 2 women, 13 men - aged 51-83 years, $\bar{x}$=67.2 years, SD=9.6 years - 1-17 years since diagnosis   Data collection:  Semi-structured interviews  Data analysis:  Content Analysis (based on Berg & Lune) | 4 themes and 13 subthemes:   1. Perceived causes, manifestations and prognosis 2. Inherent causes and external threats 3. Symptom Manifestation 4. Ambivalence between illness and normalcy 5. Ambiguity of prognosis 6. Enduring emotions 7. Feelings of negative emotions 8. Being a burden to the family 9. Trading between life and death 10. Managing the condition 11. Acceptance 12. Reviewing life successes 13. Holding on to spirituality 14. Self-care with support 15. Needs from health care professionals 16. Trust with the healthcare system 17. Information and support needs |
| 15  Kirk et al. 2018,  Denmark | People’s lived experiences of daily life and coping with recovery after Transcatheter Aortic Valve Implantation (TAVI) | Sample:   - 10 people with TAVI - 6 women, 4 men - aged 72-87 years, $\bar{x}$= 79 years - Interviews took place 3 to 4 month after implantation   Data collection:  Semi-structured interviews  Data analysis:  Ricœur’s Phenomenological Hermeneutics | 3 themes:   1. Transformation of bodily sensations 2. A changed bodily attention 3. Enhanced physical possibilities to cope with life |

**Table 2.** Continued

| Study, Year, Country | Aim/ Phenomenon under investigation | Methodology and methods | Major findings (as presented by primary authors) |
| --- | --- | --- | --- |
| 16  Li et al. 2018,  Taiwan | The self‐care coping processes among people with CHF | Sample:   - 27 people with CHF - 10 women, 17 men - aged 34-79 years, $\bar{x}$=78 years - 1-11 years since diagnosis, $\bar{x}$=3.4 years   Data collection:  Semi-structured interviews  Data analysis:  Graneheim and Lundman’s Qualitative Content Analysis | 3 themes and 11 Subthemes:   1. Responding to CHF self-care 2. Dealing with negative emotions 3. Accepting reality 4. Struggling between a self-care regime and personal preference 5. Finding ways to live with CHF 6. Enhancing understanding and knowledge about CHF 7. Maintaining outer and inner self 8. Engaging positively/negatively with other 9. Relying on religious thoughts and seeking consultation 10. Reinterpreting CHF and performing meaning-oriented coping 11. Re-evaluating the meaning of life 12. Assigning a new perspective for CHF 13. Discovering a deeper meaning behind it |
| 17  Ketilsdottir et al. 2019, Iceland | Survivors’ experiences, needs and concerns following Sudden Cardiac Arrest (SCA) and resuscitation | Sample:   - 7 male SCA survivors - aged 50-54 years - Interviews took place 9 to 24 months after SCA   Data collection:  Semi-structured interviews  Data analysis:  Interpretative Phenomenology | 5 themes:   1. Feelings of insecurity and the need for support 2. Striving to regain former life 3. Emotional challenges 4. Responding to symptoms 5. New view on life |

**Table 2.** Continued

| Study, Year, Country | Aim/ Phenomenon under investigation | Methodology and methods | Major findings (as presented by primary authors) |
| --- | --- | --- | --- |
| 18  Ryan et al. 2009,  Ireland | To describe people’s experiences of living with advanced HF | Sample:   - people with advanced HF, NYHA class III–IV - 6 participants with idiopathic ischaemic heart disease, 2 participants with cardiomyopathy, 1 participant with primary heart valve disease - 3 women, 6 men - aged 54–82 years (male participants) and 67–79 years (female participants), $\bar{x}=$68.9 years   Data collection:  Unstructured interviews  Data analysis:  Hermeneutic Phenomenology (e.g. Gadamer) | 4 themes and 9 subthemes:   1. Living in the shadow of fear    1. Night terrors    2. A precarious 2. Running on empty    1. Out of energy    2. Out of hope 3. A restricted life    1. Can’t do    2. Do it for me    3. Doing time   (4) Battling the system   1. Sorry but it's me AGAIN 2. Who are you again? |
| 19  Forman et al. 2018,  Canada | To explore people’s experiences of living with a subcutaneous ICDw including the decision-making process, implant, and follow-up care processes | Sample:   - 5 participants had previously experienced CA, and 4 participants had previously had a transvenous ICD implanted - 2 women, 13 men - aged 20–68 years   Data collection:  Semi-structured interviews  Data analysis:  Interpretive description with concurrent, Thematic Analysis | themes and 10 Subthemes:   1. Influences on decision-making    1. Physician expertise    2. Family obligations    3. Knowledge about SICD 2. Unmet education needs 3. Physical impact    1. Adapting to physical presence of device    2. Participation in physical activities 4. Psychological impact 5. Fear/anxiety 6. Gratitude 7. Acceptance 8. Recommendations    1. Patient-centered care    2. Peer support |

**Table 2.** Continued

| Study, Year, Country | Aim/ Phenomenon under investigation | Methodology and methods | Major findings (as presented by primary authors) |
| --- | --- | --- | --- |
| 20  Wood et al.2007,  USA | To explore people’s experiences living with Supraventricular Tachycardia (SVT) | Sample:   - 25 people with SVT - 5 women, 10 men - aged 18-81 years, $\bar{x}$=40 years   Data collection:  Semi-structured interviews  Data analysis:  Grounded Theory | 1 core catagory and 4 categories:   1. struggling to be believed 2. Living with the uncertainty of the occurrence and duration of an episode 3. Covering up to manage symptoms and appear normal 4. Searching for causative factors to prevent further episodes 5. Experimenting with management techniques to shorten or stop episodes |
| 21  Hasankhani et al. 2014,  Iran | To explore subjective experiences after angioplasty | Sample:   - 15 participants - with stent placement: 10 participants - without stent placement: 5 participants - 4 women, 11 men - aged 37-70 years, $\bar{x}=$55.8 years   Data collection:  Semi-structured interviews  Data analysis:  Phenomenological study design, qualitative Content Analysis | 3 core themes and 6 Subthemes:   1. Angioplasty is a milestone in the patient’s life 2. Symptom free—disease free 3. Redeﬁning life 4. Living with a mended heart 5. A sense of signiﬁcant loss 6. Feelings of vulnerability 7. Psychological distress as an integral part of the patient’s life    1. Persistent anxiety    2. Giving up. |

**Table 2.** Continued

| Study, Year, Country | Aim/ Phenomenon under investigation | Methodology and methods | Major findings (as presented by primary authors) |
| --- | --- | --- | --- |
| 22  Smith et al. 2017,  UK | To explore the experience of positive adjustment following a heart attack | Sample:   - 10 men attending a cardiac rehabilitation programme - aged 41–85 years, $\bar{x}=63.5$ years   Data collection:  Semi-structured in-depth interviews  Data analysis:  Interpretative Phenomenological Analysis | 1 overarching theme and 6 subthemes:   1. I was in control of it from the start 2. Personal resilience and adaptive coping strategies 3. Empowered experience of care and recovery 4. Responsibility to others: appreciation of social support 5. Limited impact on self despite shock of heart attack 6. ‘A new lease of life’: heart attack as trigger for positive change   (2) Acceptance of continued adjustment in relationship with body |
| 23  Banner et al. 2011,  UK | Explore women’s experiences of Coronary Artery Bypass Graft surgery | Sample:   - 30 women having undergone CABG - Interviews took place before surgery, 6 weeks and 6 months after surgery - aged 53-80 years, $\bar{x}$=68.2 years   Data collection:  Semi-structured interviews  Data analysis:  Grounded Theory | 1 Core category and 16 categories, reflecting the recovery stages during which the interviews took place:  Core Category: Normality   1. Preserving normality    1. Symptom recognition and translation 2. Disrupted normality (Preoperative interviews)    1. Help seeking    2. Diagnosis and referral    3. Conceptualising surgery    4. Living with CHD    5. Waiting for CABG surgery 3. Relinquished normality (Early recovery interviews)    1. Immediate postoperative recovery    2. Early recovery    3. Support postoperatively    4. Resuming domestic functioning    5. Establishing boundaries 4. Renegotiated normality (Long-term recovery interviews)    1. Recovering    2. Renegotiating normal    3. Cardiac rehabilitation    4. Long-term support    5. Evaluating outcome |

**Table 2.** Continued

| Study, Year, Country | Aim/ Phenomenon under investigation | Methodology and methods | Major findings (as presented by primary authors) |
| --- | --- | --- | --- |
| 24  Malm et al. 2006,  Sweden | To examine the experiences of daily living of people with a pacemaker | Sample:   - 13 people living with pacemakers - 7 women, 6 men - aged 22–82 years, $\bar{x}=59.2$ years   Data collection:  Semi-structured interviews  Data analysis:  Grounded Theory | Combinations of 2 core categories ((1) social participation and (2) emotional status) lead to 4 categories: Feeling overprotected, feeling inadequate, imposing restrictions, and recapturing life |
| 25  Strömbäck et al 2018, Sweden | To describe people’s experiences of suffering a second MI | Sample:   - 8 MI survivors - 2 women, 6 men - aged 49–79 years   Data collection:  Semi-structured interviews  Data analysis:  Qualitative Content Analysis | 1 theme and 4 categories:   1. Realizing the seriousness 2. Knowledge from previous experience 3. A wake-up call for lifestyle changes 4. The future becomes unpredictable 5. Trying to ﬁnd balance in life |

**Table 2.** Continued

| Study, Year, Country | Aim/ Phenomenon under investigation | Methodology and methods | Major findings (as presented by primary authors) |
| --- | --- | --- | --- |
| 26  Brännström et al. 2006, Sweden | To illuminate the meaning of living with severe CHF in palliative advanced home care through people’s narratives | Sample:   - 4 people with severe CHF - 1 woman (NYHA III), 3 men (NYHA IV) - aged 72 - 81 years   Data collection:  Narrative interviews  Data analysis:  Phenomenological–Hermeneutic method inspired by Ricœur | 4 themes and 12 sub-themes:   1. Being aware that one´s life hangs by a fine thread 2. Sensing one’s bad heart 3. Visiting death’s door 4. Being reminded about one’s failing health by physiological measurements 5. Struggling to cope with one’s unpredictable deteriorated body 6. Facing difficulties to keep one’s weight stable 7. Facing varying degrees of laborious shortness of breath 8. Facing varying degrees of laborious pain in addition to that in the heart 9. Facing difficulties standing on one’s legs and walking 10. Facing laborious fatigue 11. Struggling with isolation 12. Feeling lonely 13. Being trapped at home 14. Being positively dependent on receiving care that facilitates life at home 15. Feeling secure when receiving palliative advanced home care 16. Managing to be technically and pharmacologically dependent at home |
| 27  Petriček et al. 2017, Croatia | To explore the experiences of life with MI, the challenges people face during  the process of accepting their condition, and the setting and resetting of their personal goals | Sample:   - 30 people with a primary diagnosis of MI - 14 women, 16 men - Age range is not reported, $\bar{x}=$53.6 years - All participants reported one or more co-morbidities; the most frequent was hypertriglyceridemia (30/30), followed by hypertension (24/30), and diabetes mellitus type 2 (18/30)   Data collection:  Semi-structured interviews  Data analysis:  Grounded Theory | 3 main themes and 17 sub-themes:   1. A good adjustment—the ‘new normality’ 2. Disease is just a normal part of my life, just another obligation to think   about   1. My body is different but still functional 2. I managed to keep something valuable from my ‘past’ life 3. I even gained some benefits 4. I gained self-esteem by creating a balance between myself and the disease 5. Taking control of my life 6. Disease is my life partner 7. Maladjustment—a continuous search for the ‘new normality’ 8. Struggling against MI 9. MI makes me anxious 10. It’s just abnormality in diagnostic tests 11. Perceived needs in the search for the ‘new normality’ 12. Overcoming the anxiety of MI recurrence‘ 13. Need for information 14. Timeline 15. Patience and steadiness 16. Objective and subjective health state improvement 17. Taking control over the disease 18. Social network support |

**Table 2.** Continued

| Study, Year, Country | Aim/ Phenomenon under investigation | Methodology and methods | Major findings (as presented by primary authors) |
| --- | --- | --- | --- |
| 28  Presciutti et al. 2022, USA | To better understand CA survivorship challenges, and to identify ways to improve CA survivorship. The study was part of a larger study exploring patient-centered outcomes after CA | Sample:   - 15 CA survivors - 73% (n=11) of survivors reported experiencing an OHCA, 87% (n=13) received an ICD, 6.7% (n=1) used a wearable ICD, and 47% (n=7) reported receiving targeted temperature management - 8 women, 7 men - aged 34-71 years   Data collection:  Semi-structured one –to-one interviews  Data analysis:  Thematic Analysis, using the Framework Method | 2 main domains of investigation   1. Challenges with the overarching theme of ‘Feeling unprepared’ to confront survivorship: Lack of resources for treating extracardiac symptoms, Little education/knowledge of condition, Lack of accurate expectations for recovery, Social challenges, Difficulty returning to work, Difficulty managing medications, Feeling abandoned by providers, Lack of emotional support, Difficulty adapting to new normal 2. Recommendations with the three subdomains    1. Systemic: Provide resources to manage extracardiac symptoms, Provide appropriate expectations and education about cardiac arrest survivorship, Educate providers about cardiac arrest survivorship, Follow-up with survivors, Include caregivers in treatment planning whenever possible    2. Social: Attend peer support groups, Spend time with family and friends, Provide emotional support resources for family and caregivers    3. Individual: Acceptance, Resilient coping, Regain control in life, Seek treatment for extracardiac symptoms, Focus on meaning and purpose |
| 29  Salminen-Tuomaala et al. 2012, Finland | To describe factors that influence people’s coping with MI during hospitalization. | Sample:   - 28 people recovering from MI - 12 women, 16 men - aged 32 - 82 years   Data collection:  Theme interviews  Data analysis:  Grounded Theory | The description of 1 core category, “Coping with myocardial infarction: Seeking lost control”, is followed by an illustration of the 2 related main categories and 3 subcategories and their interrelations.   1. Core: “Coping with myocardial infarction: Seeking lost control”    1. Main Category 1: Factors supporting the patients coping    2. Main Category 2: Factors interfering with patients coping 2. Subcategory: Patient -related factors 3. Subcategory: Condition-related factors 4. Subcategory: Interaction-related factors |

**Table 2.** Continued

| Study, Year, Country | Aim/ Phenomenon under investigation | Methodology and methods | Major findings (as presented by primary authors) |
| --- | --- | --- | --- |
| 30  Inyom et al. 2020,  Germany | To describe and understand the lived experiences of people undergoing long-term circulatory  support with Left-Ventricular Assist Device (LVAD) | Sample:   - 21 people living with LVAD - 7 women, 14 men - aged 37-78 years, $\bar{x}$=61 years - median time since implantation 26 months   Data collection:  Semi-structured interviews  Data analysis:  Phenomenological method by Colaizzi | 8 themes, each with effect on QOL:   1. Whether they had experienced improvements or setbacks in their recent health condition 2. Experiencing burdens from their device such as weight and handling 3. Limitations in their physical ability such as participating in sports, their inability to work or reduced sleep 4. Reduced social interactions 5. Reduction in sexual activity and performance 6. Experiencing emotional and psychological problems and experiencing anxiety 7. Support from family and friends 8. Optimism and obtaining “a second chance” at life |
| 31  Dehghanzadeh et al. 2018,  Iran | To explore the process of living with Cardiac Resynchronization Therapy Defibrillator (CRT-D) | Sample:   - 17 people being treated with CRT-D for HF - 6 women, 13 men - aged 25-93 years, $\bar{x}$=58.94 years, SD=15.93 years - CRT-D implantations took place 1-108 months prior to the interviews, $\bar{x}$=25.94 months, SD=33.10 months   Data collection:  Semi-structured interviews  Data analysis:  Grounded Theory | 1 core category and theory of 3 phases: Core Category: doubtful accepting  1. Phase: losing integrity   1. Perceiving cardiac resynchronization therapy defibrillator as threat 2. Endangered physical comfort 3. Altered physical self   2. Phase: attempting to cope with CRT-D   1. Resorting to spirituality 2. Stress management 3. Information Seeking and peer comparing   3. Phase: coexisting   1. From frustration to empowerment 2. Barriers and facilitators to living with cardiac resynchronization therapy defibrillator |

**Table 2.** Continued

| Study, Year, Country | Aim/ Phenomenon under investigation | Methodology and methods | Major findings (as presented by primary authors) |
| --- | --- | --- | --- |
| 32  Heo et al. 2009,  USA | To explore the perceptions of quality of life (QOL) from the perspective of people with HF | Sample:   - 20 people with HF - 6 women, 14 men - Age range is not reported, $\bar{x}$=58 years, SD=10 years - NYHA class II-IV   Data collection:  Semi-structured interviews  Data analysis:  Content Analysis | Different Aspects of QOL from Patient’s perspectives:  Definition of QOL:   1. Ability to perform physical and social activities 2. Maintaining happiness 3. Engaging in fulfilling relationships   Factors affecting QOL:   1. Health status 2. Psychological factors 3. Economic status 4. Social factors 5. Spirituality 6. Health related behavior |
| 33  Bhattacharyya et al. 2015,  UK | To explore between-ethnicity and within-ethnicity variation in adaptation, and the psychological impact of an acute cardiac event among UK South Asian and white British people | Sample:   - 28 people with MI, angina or acute arrhythmia - 5 women, 23 men - aged 44-88 years, $\bar{x}$=66.6 years, SD=12.2 years - ethnically diverse (Asian, Caucasian)   Data collection:  Semi-structured Interviews  Data analysis:  Framework Analysis | 5 themes:   1. Psychological Impact:    1. Emotional sequelae—low mood, anxiety and fear    2. Changing role perception of self and how others perceive 2. Perceived physical impact of CHD 3. Attitudes to the future 4. Social and professional support    1. Family/friends    2. Professional service support: National Health Service: GP/hospital, Cardiac rehabilitation programme 5. Practical adaptations made after the event    1. Return to work    2. Lifestyle changes |

**Table 2.** Continued

| Study, Year, Country | Aim/ Phenomenon under investigation | Methodology and methods | Major findings (as presented by primary authors) |
| --- | --- | --- | --- |
| 34  Surikova et al. 2020, Canada | To examine cultural and gender-based influences on QOL among people with CHF | Sample:   - 30 people with CHF - 10 women, 20 men - Age range is not reported, at least 19 years old, $\bar{x}$=54.6 years - time since diagnosis: $\bar{x}$=8.3 years - NYHA class I-III - ethnically diverse (Black, Chinese, South Asian, Caucasian)   Data collection:  Semi-structured interviews  Data analysis:  Inductive qualitative approach with Thematic Content Analysis | 5 key themes   1. CHF as an emergent reality 2. Quality of life and disruption of life course milestones 3. The challenge to accept CHF and re-evaluation of quality of life 4. Impact on social activities essential to quality of life 5. Life with CHF as a commitment to culturally tailored self-care |
| 35  Paturzo et al. 2016, Italy | To describe the lived experience of adults with HF | Sample:   - 30 people with HF - 10 women, 20 men - At least 3 months since diagnosis - NYHA class I-IV - aged 48-86 years, $\bar{x}$=71 years, SD=9.15 years   Data collection:  Unstructured interviews  Data analysis:  Hermeneutic Phenomenology (based on Cohen) | 7 themes:   1. Important life changes 2. Social isolation caused by the illness 3. Anger and resignation associated with the disease 4. Relief from spirituality 5. Will to live 6. Uncertainty about the future 7. The inescapability of disease and death |

**Table 2.** Continued

| Study, Year, Country | Aim/ Phenomenon under investigation | Methodology and methods | Major findings (as presented by primary authors) |
| --- | --- | --- | --- |
| 36  Altiok et al. 2015,  Turkey | To determine the lived experiences of people with Atrial Fibrillation (AF) | Sample:   - 32 people with AF - 16 women, 16 men - Age range is not reported, $\bar{x}$=66.9 years, SD=7.9 years - At least 6 months since diagnosis   Data collection:  Semi-structured interviews  Data analysis:  Phenomenological method by Colaizzi | 4 Themes, 16 subthemes:   1. Patient’s mental status regarding the disease    1. Acceptance of the disease    2. Feelings of burnout due to frequent visits to hospital for follow-up appointments    3. Dependence    4. Reaction    5. Fatalism    6. Anxiety    7. Fear of being paralyzed 2. Patient’s social status regarding the disease    1. The disease has adverse effects on social life    2. The disease has adverse effects on ﬁnancial situation    3. Protective-caring behavior of family members 3. Patient’s physical condition regarding the disease    1. Inability to carry out daily living activities related to the disease    2. Sexual Problems related to the disease 4. Disease management and coping with the disease    1. Feelings of insecurity due to frequent change of healthcare providers    2. Negative coping with having the disease due to lack of information    3. Positive coping with living with atrial fibrillation |
| 37  Dumit et al. 2016,  Lebanon | To explore the cultural perceptions of cardiac illness and the associated meaning of self-care among Lebanese participants | Sample:   - 15 people with Coronary Artery Disease (CAD) - Sex/gender is not reported - At least 3 years since diagnosis - Age range is not reported, participants were at least 30 years old, $\bar{x}$=62 years   Data collection:  Semi-structured interviews  Data analysis:  Descriptive Analysis (based on Creswell, Sandelowski) | 1 overarching theme, 2 themes and 10 subthemes:  Overarching cultural theme:   1. Meaning of self-care 2. Managing their illness 3. Theological and cultural interpretation of illness    1. Thanking God    2. Relying on God    3. Accepting fate 4. Response to cardiac event    1. First thought came to mind    2. Responses to initial symptoms    3. Positive outlook toward life    4. Fear of death    5. Dissonance between life and death |

**Table 2.** Continued

| Study, Year, Country | Aim/ Phenomenon under investigation | Methodology and methods | Major findings (as presented by primary authors) |
| --- | --- | --- | --- |
| 38  Brink 2009, Sweden | This study explores people’s experiences of adaptation to MI consequences after one year, focusing on experiences of “the self”. | Sample:   - 19 people with first-time MI - 10 women, 9 men - Age range is not reported, $\bar{x}$=63.66 years, SD=10.31 years - 1 year after MI   Data collection:  Unstructured interviews  Data analysis:  Grounded Theory | 1 theoretical model, 2 core categories, 2 forms of behavior  Theoretical Model:  4 different adaptation positions:   1. Put up with current health 2. Struggle for health 3. Ignore illness 4. Struggle against illness   Core categories:   1. Self-agency 2. Coping with illness consequences   Forms of behavior:   1. Self-modifying 2. Self-protecting |
| 39  Kamphuis et al. 2004, Netherlands | To explore the lived experiences of ICD recipients during the ﬁrst year after implantation | Sample:   - 21 people living with ICD - 9 women, 12 men - aged 47-69 years, $\bar{x}$=58.3 years, SD=1.1 years - Interviews took place 1, 6, and 12 months after implantation   Data collection:  Semi-structured interviews  Data analysis:  Content Analysis (based on De Ridder / Kuckartz) | 7 major categories:   1. Physical deterioration 2. Cognitive change 3. Perceived social support 4. Dependency 5. Contact with the doctor 6. Confrontation with mortality 7. Uncertainty surrounding having a shock |

**Table 2.** Continued

| Study, Year, Country | Aim/ Phenomenon under investigation | Methodology and methods | Major findings (as presented by primary authors) |
| --- | --- | --- | --- |
| 40  MacDermott 2002,  UK | Understanding the effects of Angina Pectoris from people’s perspectives | Sample:   - 7 people with stable Angina Pectoris; 3 additional interviews were done, but discarded for methodical reason - Age is not reported - Sex/gender is not reported   Data collection:  Unstructured interviews  Data analysis:  Thematic Analysis (based on Jaspers) | 7 themes:   1. Limitations and adjustment 2. Resignation 3. Indignation 4. Caution 5. Reluctant compliance 6. Surprise 7. The unknown |
| 41  Jensen et al. 2003,  Denmark | To investigate people’s illness experiences after ﬁrst MI, focusing on the recovery process over time | Sample:   - 30 people with first-time MI - 8 women, 22 men - aged 41-80 years, $\bar{x}=59.7$ years   Data collection:  Semi-structured interviews  Data analysis:  Qualitative Content Analysis according to Polit and Hungler | Overall Theme: Uncertainty of the life situation   - - - - 1. Treatment-seeking behaviour (the pre-hospital phase)   Relieving the symptoms  Previous experiences  Impression of relatives’ opinion   - - - - 1. Existential threat   The acute phase: Close to death, Coping with the threat  The rehabilitation phase: Emotional consequences of the illness   - - - - 1. Preventing another coronary   The acute phase: Psychosocial constraints, Physiological strains, Heredity and envrionment  The rehabilitation phase: Reducing psychosocial strains, Reducing physiological strains   - - - - 1. Need for knowledge and support   Support from the lay network, Support from the professional network |

**Table 2.** Continued

| Study, Year, Country | Aim/ Phenomenon under investigation | Methodology and methods | Major findings (as presented by primary authors) |
| --- | --- | --- | --- |
| 42  Brink et al. 2006,  Sweden | To explore the self-regulation process in order to increase understanding of readjustment from a ﬁrst-time MI | Sample:   - 21 people with MI - 10 men, 11 women - aged 48-83 years - first-time infarction 5 months prior to the interviews   Data collection:  Unclear but multiple questions (conducted in 1999)  Data analysis:  Grounded Theory | One overarching theme with interconnected subthemes:   1. Reorienting the active self    1. Fatigue and other health complaints    2. Illness perception    3. Minimizing consequences    4. Sense of control    5. Moderating activities |
| 43  Keaton et al. 2000,  USA | Uncover the lived experience of cardiac therapy for men with CAD in Northwest Ohio | Sample:   - 5 men with CAD, partaking in cardiac therapy - aged 60-70 years - diagnosed at least 2 years prior to the interviews - 4 MI, 1 sudden cardiac death   Data collection:  Unstructured interview  Data analysis:  Phenomenological method by Colaizzi | 8 interconnected themes:   1. Feeling of fear and disbelief 2. Being aware of mortality 3. Losing control of self 4. Halting of usual activities and future plans 5. Heightening awareness of spirituality and belief in a higher power 6. Resigning to acceptance of external forces and required change 7. Increasing sense of well-being 8. Receiving a second chance and moving forward |
