# Supplementary material for: “That kind of changes things”: a meta-synthesis of the lived experiences of people with chronic heart disease
Source: Health Qual Life Outcomes. 2025 Sep 18;23:84. doi: 10.1186/s12955-025-02423-6 (PMC12447608; doi:10.1186/s12955-025-02423-6)
Supplement: Supplementary file 3 — Supplementary Material 3. [file 12955_2025_2423_MOESM3_ESM.docx]

**Additional File 3**

**Table 1.** Example quotes subsumed under the theme ‘I feel no longer safe in my body’

| Quote | Code |
| --- | --- |
| Having this thing totally wrecked my life. I felt so scared and helpless because I never knew when I’d have another episode, how bad that episode would be, if this new drug would work…because many of them didn’t…. or if it never stopped racing…. would I die?^1(p.6)^ | I could die at any time |
| I think it makes me realize that at any time I might die.^2(p.75)^ |  |
| But...it’s only night times, sometimes I do have sleepless nights, I mean I sleep late. Because sometimes I (will) think about my condition, will it help me (or) not, or will I get another attack, that I am scared of. (Be)cause I don’t want the pain...if you are in bad...very severe pain, you know...you going to lose your life, right? (P9, female, 57 years old)^3(p.395)^ |  |
| When you get home on your own you realize, every pain you get you’re not sure if you should have it... One night I’d had a bad coughing and sneezing fit and I thought I’d pushed the bone out. I lay there thinking, ‘please God, let it go back’. It’s not knowing within yourself what’s right or wrong, you know a pain, a bit of shortness of breath... It’s those sort of things that wouldn’t happen if you knew a bit more... I suppose I didn’t know what was normal^4(p.924)^ | I'm extremely insecure about bodily processes |

**Table 2.** Example quotes subsumed under the theme ‘Suddenly, I have less of a future’

| Quote | Code |
| --- | --- |
| In the past, I would never think about having a premature death, but now I am getting to realize it is possible. (A 48-year-old male patient)^5(p.5)^ | My future is uncertain |
| One participant said ‘Who knows how many days I have to live?’ and ‘You live with this big question mark.’ Another patient stated ‘I know what happened yesterday or today but I don’t know what could happen tomorrow. I try to live my life without thinking about my future, but sometimes there grows up inside of me a deep sense of uncertainty.^6(p.268)^ |  |
| One, one, one wants willingly...as a human that, “Well, now one is ill. Now it was all fixed, then it’s all right!” This isn’t something like that! This feebleness that one has afterwards is very paralyzing. It’s...when we are sitting like this, I don’t notice very much of it, you know...but just to move yourself physically gives you, all the time, a reminder of, “Yes, but you aren’t entirely well! You will probably never be well! Your heart is damaged! You, you, you have that handicap.” And that...I’m like that, it’s not much fun then. And, and I suppose that’s a part of the anxiety then, that, yes you are...not completely...in your prime. And that’s, that’s also one of those things that...lie behind the anxiety.^7(p.332)^ | My options for the future are limited |
| Well I thought it was a disaster I thought I was going to be stuck there ‘til I was 65 and have a nice easy happy retirement and then when I had the heart attack I said woooaaahhh I’m away to the mixers (laughs) no easy retirement. Well I mean I always wanted to work long enough to retire’.^8(p.269)^ |  |

**Table 3.** Example quotes subsumed under the theme ‘My identity feels shattered’

| Quote | Code |
| --- | --- |
| I think that I've become a very old man when I see myself in the mirror. I don't like it [laughs]^9(p.1237)^ | The heart condition makes me feel old |
| Well, I’m not the same person anymore. in the past people would repair their broken china dishes, but didn’t use them as they would break easily. They would look after it. I’m just like it (a broken china dish). (A 70-year-old male patient; p6)^5(p.145)^ | I feel useless |
| I work in construction; I used to go to work from morning till 5 to 6 in the afternoon, but not anymore. I am not the same person; neither my physical condition nor the fatigue let me anymore. (A 54-year-old male patient; p11)^5(p.147)^ | I can no longer be as productive as I used to be |
| This disease is affecting my social life. I cannot go anywhere. I cannot move anything. I cannot prepare food for my friends and it is not appropriate to have guests without offering them food. I used to visit my friends at their places and I would have them in my house. We had regular home meetings. What a hardworking woman I was! But, for the last two years now, I haven't been able to do any of it because of this disease. My heart beats really fast when I have palpitations or when I dream, so I try having a rest. I love shopping, hanging around in shopping malls or markets, or spending money, but I cannot go to these places myself. I have to have somebody with me all the time. (67 years old, female)^10(p.308)^ | Depending on the help of others |

**Table 4.** Example quotes subsumed under the theme ‘My disease strains my relationships’

| Quote | Code |
| --- | --- |
| One man noted ‘My friends do not call me and I feel so isolated from the world because I stay home and I cannot move and talk to anyone.^6(p.267)^ | Living with heart disease can be isolating |
| You go through 3 months’ worth of rehab and it’s almost like you’re almost like dropped like a hot potato. It’s like, yeah, you completed your program but there’s no follow-up after that to say, hey, 3 months later, how are you doing?^11(p.6)^ | Feeling abandoned by healthcare professionals |
| It was a very challenging time for our marriage and not only for me being the victim of it, but my wife had a whole other host of feelings because she was the person that came to my aid when I went down and gave me cardiopulmonary^11(p.7)^ | Being a burden to others |
| If I go to Stockholm then I’m a burden on my children. I’ve felt this the whole time. I want to manage on my own as far as I can (woman, aged 79)^12(p.372)^ |  |
| My family, they try to [be] extra sensitive to things. So like there will be times, for example, they won’t invite me to an event or something like that thinking that it’s too late, …[I] would be too tired…, you know that sort of thing, or “it’s too much, we won’t invite her husband to go along for the weekend, the guys’ weekend trip, because, you know, he’s not gonna wanna leave her for the whole weekend”. But for us we say, “let us be the ones to say no”…. Because they are super sensitive, sometimes they forget that sometimes we just want to be normal…. ID 14, [Caucasian], Female, 33 years old^13(p.4/5)^ | Being overprotected by others |

**References**

1. Wood KA, Wiener CL, Kayser-Jones J. Supraventricular tachycardia and the struggle to be believed. Eur J Cardiovasc Nurs 2007;6:293-302. doi: 10.1016/j.ejcnurse.2007.02.006
2. Keaton KA, Pierce LL. Cardiac therapy for men with coronary artery disease: the lived experience. J Holist Nurs. 2000;18(1):63-85
3. Seah ACW, Tan KK, Huang Gan JC, Wang W. Experiences of patients living with heart failure: A descriptive qualitative study. J Transcult Nurs. 2016;27(4):392-399. doi:10.1177/1043659615573840
4. Banner D, Miers M, Clarke B, Albarran J. Women's experiences of undergoing coronary artery bypass graft surgery. J Advanced Nurs 2012;68:919-930. doi: 10.1111/j.1365-2648.2011.05799.x
5. Hasankhani H, Gholizadeh L, Mohammadi E, Zamanzadeh V, Allahbakhshian A, Ghaffari S, Allahbakhshian M. The lived experiences of patients post coronary angioplasty: A qualitative study. J Vasc Nurs. 2014;32(4):144-150. doi:10.1016/j.jvn.2014.04.001
6. Paturzo M, Petruzzo A, Bertò L, Cohen MZ, Alvaro R, Vellone E. The lived experience of adults with heart failure: a phenomenological study. Ann Ig. 2016;28(4):263-273. doi:10.7416/ai.2016.2105
7. Bremer A, Dahlberg K, Sandman L. To Survive Out-of-Hospital Cardiac Arrest: A Search for Meaning and Coherence. Qual Health Res. 2009;19(3):323-338. doi:10.1177/1049732309331866
8. MacDermott AFN. Living with angina pectoris—a phenomenological study. Eur J Cardiovasc Nurs. 2002;265-272
9. Kirk BH, De Backer O, Missel M. Transforming the experience of aortic valve disease in older patients: A qualitative study. J Clin Nurs. 2019;28:1233-1241. doi:10.1111/jocn.14732
10. Altiok M, Yilmaz M, Rencüsoğullari I. Living with Atrial Fibrillation: An Analysis of Patients' Perspectives. Asian Nurs Res 2015;9:305-311. doi: 10.1016/j.anr.2015.10.001
11. Presciutti A, Siry-Bove B, Newman MM, Elmer J, Grigsby J, Masters KS, Shaffer JA, Vranceanu AM, Perman SM. Qualitative Study of Long-Term Cardiac Arrest Survivors' Challenges and Recommendations for Improving Survivorship. JAHA 2022;11:e025713. doi: 10.1161/JAHA.121.025713
12. Kristofferzon ML, Löfmark R, Carlsson M. Managing consequences and finding hope—experiences of Swedish women and men 4-6 months after myocardial infarction. Scand J Caring Sci. 2008;22(3):367-375. doi:10.1111/j.1471-6712.2007.00538.x
13. Surikova J, Payne A, Miller KL, Ravaei A, Nolan RP. A cultural and gender-based approach to understanding patient adjustment to chronic heart failure. Health Qual Life Outcomes. 2020;18(1):238. doi:10.1186/s12955-020-01482-1
